# Initial Steps to Engineer Coproheme Decarboxylase to Obtain Stereospecific Monovinyl, Monopropionyl Deuterohemes

**DOI:** 10.3389/fbioe.2021.807678

**Published:** 2022-01-24

**Authors:** Hanna Michlits, Nina Valente, Georg Mlynek, Stefan Hofbauer

**Affiliations:** ^1^ Department of Chemistry, Institute of Biochemistry, University of Natural Resources and Life Sciences, Vienna, Austria; ^2^ Core Facility Biomolecular and Cellular Analysis, University of Natural Resources and Life Sciences, Vienna, Austria

**Keywords:** coproheme decarboxylase, tyrosyl radical, stereospecificity, porphyrin synthesis, redox enzyme

## Abstract

The oxidative decarboxylation of coproheme to form heme *b* by coproheme decarboxylase is a stereospecific two-step reaction. In the first step, the propionate at position two (p2) is cleaved off the pyrrole ring A to form a vinyl group at this position. Subsequently, the propionate at position four (p4) on pyrrole ring B is cleaved off and heme *b* is formed. In this study, we attempted to engineer coproheme decarboxylase from *Corynebacterium diphtheriae* to alter the stereospecificity of this reaction. By introducing a tyrosine residue in proximity to the propionate at position 4, we were able to create a new radical center in the active site. However, the artificial Tyr183^•^ radical could not be shown to catalyze any decarboxylation.

## Introduction

**SCHEME 1:**
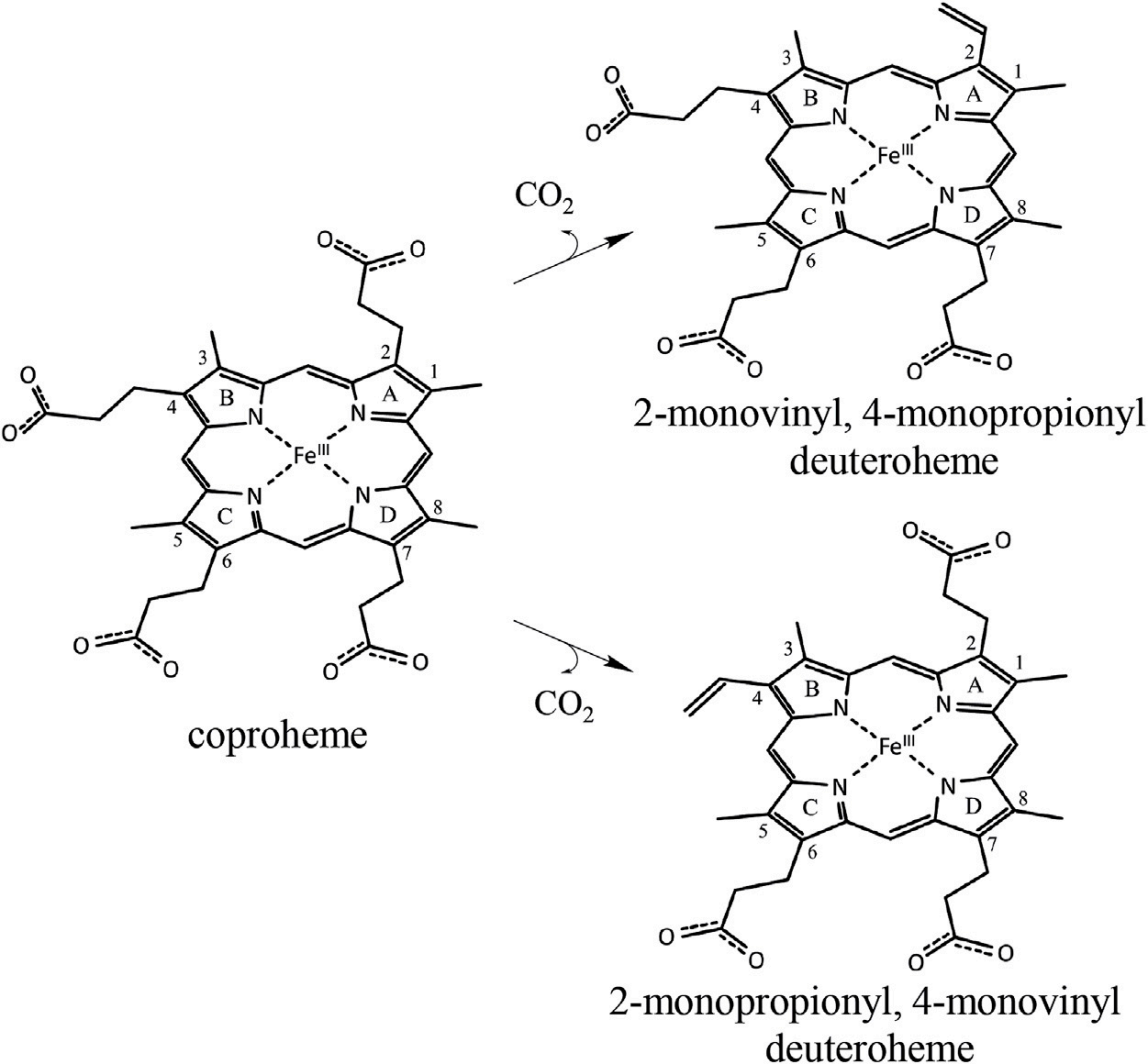
Coproheme is converted to the reaction intermediate 2-monovinyl-4-monopropionyl deuteroheme during the first step of the ChdC reaction. Due to the active site architecture and conserved coproheme binding orientation, p2 is cleaved off first by a radical attack of the catalytic Y135 before a reorientation of MMD and decarboxylation of p4. Introducing a Tyr residue at the site of p4, we hope to introduce a second active site and achieve decarboxylation of p4 independently of the reorientation of MMD. In a double variant, lacking the original Y135 would theoretically yield the intermediate product 2-monopropionyl-4-monovinyl deuteroheme.

Specific production of different porphyrins is usually performed by organic synthesis but can be, for some isomers and targets, very costly and labor intensive (Lash et al., 1999; Lash et al., 2010; Lash et al., 2011; Lash, 2016). In this work, we test the possibility to use engineered coproheme decarboxylase (ChdC) for the production of stereospecific three-propionate intermediates, namely, 2-monovinyl-4-monopropionyl deuteroheme (MMD) and 2-monopropionyl-4-monovinyl deuteroheme (sometimes referred to as harderoheme III and harderoheme IV, respectively) (Scheme 1).

**SCHEME 2:**
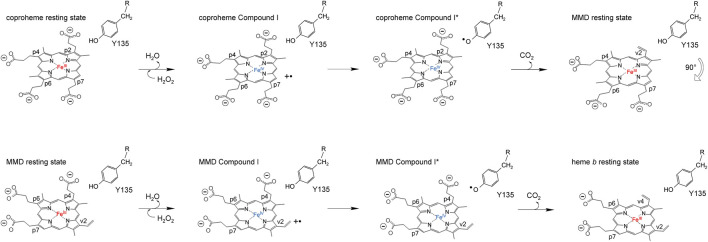
Proposed reaction mechanism of coproheme decarboxylation to MMD (top row) and MMD after the 90° reorientation to heme *b* (bottom row).

ChdCs are enzymes involved in the prokaryotic heme biosynthesis pathway mainly of monoderm bacteria (Dailey et al., 2015; Dailey et al., 2017), but some diderm or intermediate representatives also use this pathway (Pfanzagl et al., 2018). They catalyze the hydrogen peroxide–driven oxidative decarboxylation of iron coproporphyrin III (coproheme) to iron protoporphyrin IX (heme *b*) (Celis et al., 2015; Hofbauer et al., 2016). The conversion of coproheme to heme *b* requires two consecutive decarboxylation steps that are facilitated by redox reactions. This reaction is started by the addition of an oxidant (e.g., hydrogen peroxide or chlorite). After initial oxidation of coproheme to Compound I (two-electron deficient reaction intermediate), a catalytic tyrosine radical is generated, which is completely essential for both decarboxylation reactions (Streit et al., 2018; Milazzo et al., 2019). The tyrosine radical initiates cleavage of a CO_2_ molecule from the porphyrin substituent and the formation of a vinyl group by a hydrogen atom abstraction of the β-carbon of the respective propionate (Celis et al., 2017; Hofbauer et al., 2021). Mechanistic studies revealed the order of decarboxylation and proved that propionate at position 2 (p2) is decarboxylated first. After reorientation of the three-propionate intermediate, a second decarboxylation cycle is initiated by another oxidant molecule to cleave off the propionate group at position 4 (p4) (Milazzo et al., 2019; Michlits et al., 2020) (Scheme 2). In order to unravel the complete reaction cycle of this highly interesting enzymatic system, it is also necessary to investigate the second part of this redox reaction. The three-propionate intermediate would enable to follow oxidation of the ferric monovinyl, monopropionyl deuteroheme to the corresponding Compound I species by biophysical and biochemical methods.

In this work, we present rationally designed *Cd*ChdC variants, which are supposed to pave the way for the stereospecific production of three propionate intermediates. In our previous work, we presented that a variant (H118F) of the actinobacterial coproheme decarboxylase from *Cornyebacterium diphteriae* (*Cd*ChdC) accumulates 2-monovinyl-4-monopropionyl deuteroheme, which is the natural intermediate of the native decarboxylation reaction. H118, unique in actinobacterial ChdCds, is part of a flexible loop, linking the N- and the C-terminal domain of one subunit in ChdCs (Hofbauer et al., 2021). In this variant, the catalytic tyrosine (Y135) of *Cd*ChdC is positioned correctly to facilitate decarboxylation of p2 but due to steric hindrance, the reorientation of the three-propionate intermediate is prohibited and therefore no decarboxylation of p4 occurs (Sebastiani et al., 2021). In this study, we designed variants where another tyrosine is introduced at the site of p4 (W183Y). This potentially generates a second radical site and rotation-independent decarboxylation of p4 or a double variant (Y135A/W183Y) replacement of the original radical site and thereby reversing the decarboxylation reaction. This double variant cannot cleave off p2, but if a tyrosyl radical is formed at the newly introduced Y183, the first precondition is achieved to stereospecifically produce 2-monopropionyl-4-monovinyl deuteroheme. We produced, expressed, and characterized relevant *Cd*ChdC variants and studied their catalytic potential by spectroscopic methods and mass spectrometry. Also, we succeeded in solving the crystal structure of the W183Y variant, which allows evaluating the steric restraints around the newly introduced tyrosine residue.

## Materials and Methods

### Cloning, Expression, and Purification of Wild-Type *Cd*ChdC and Variants

As previously described in detail (Michlits et al., 2020), the *Cd*ChdC gene (DIP1394) was synthesized and cloned into a pD441-NH vector (ATUM, Newark, California). We complemented the pD441-NH vector with an HRV 3C protease cleavage site between the N-terminal His-tag and the protein of interest. Single and multiple variants of *Cd*ChdC were produced by site-directed mutagenesis using the QuikChange Lightning Kit (Agilent Technologies).

The plasmids were transformed into electro-competent *E. coli* Tuner (DE3) (Merck/Novagen, Darmstadt, Germany) cells for recombinant protein production. Expression cultures of 500 ml (LB medium 100 μg ml^−1^ Kanamycin) were inoculated with 1 ml overnight culture. After 3 h at 37°C and 180 rpm shaking, the cultures were cooled to 16°C prior to induction with isopropyl-β-d-thiogalactopyranoside (IPTG, 0.5 mM) and kept in cultivation for expression overnight. Cells were harvested by centrifugation (4°C, 2700 *g*, 30 min) and pellets were frozen at −30°C.

Cell lysis was performed by resuspending the pellets from 500 ml culture volume in 50 ml lysis buffer (LB: 50 mM phosphate buffer pH 7.4, with 500 mM NaCl, 5% glycerol, and 0.5% Triton X-100) and ultrasonication (for 1 min pulsed 1 s of sonication with 1 s between pulses, 90% power) on ice. The lysate was cleared by centrifugation (4°C, 38,720 *g* for 30 min) and filtration (0.45 µM pore size filter), before loading it on a His-trap fast flow affinity column (5 ml, GE Healthcare) preequilibrated with binding buffer (BB: 50 mM phosphate buffer, pH 7.4, with 500 mM NaCl). The column was flushed with BB and equilibrated with cleavage buffer (CB: 50 mM Tris-HCl with 150 mM NaCl and 1 mM EDTA). Column cleavage with the His-tagged HRV 3C PreScission Protease was done overnight at 4°C after manually loading the protease to the column with a syringe.

The eluate was concentrated using a centrifugal filter unit (50 kDa cutoff, Amicon Ultra-15, Merck Millipore Ltd., Tullagreen, Carrigtwohill Co. Cork, Ireland) by centrifuging at 4,500 *g* for 10–20 min in order to attain a volume of 1–3 ml. Protein for enzymatic activity assays was further rediluted in storage buffer (SB: 100 mM phosphate buffer pH 7.4, with 100 mM NaCl) and reconcentrated in the centrifugal unit, repeating this step 2–3 times. Protein used for crystallization was further purified by size exclusion chromatography (SEC) using a HiLoad 16/600 Superdex 200 pg, GE Healthcare column equilibrated with SB.

### Decarboxylation Activity of Wild-Type *Cd*ChdC and Variants Followed by UV–Vis Spectroscopy and Mass Spectrometry

As previously described (Michlits et al., 2020), H_2_O_2_ induced conversion of coproheme to heme *b* was investigated by analyzing the same reaction mix in UV–Vis absorbance and by mass spectrometry. Typically, 1,000 µL enzyme solution in reaction buffer (RB: 50 mM phosphate buffer, pH 7) with around 15 μM apoenzyme and 10 μM coproheme was titrated with subequimolar amounts of hydrogen peroxide (4 mM H_2_O_2_ stock in RB) in a Cary 60 spectrophotometer (Agilent Technologies). About 10 µl samples from this reaction mix were drawn and directly taken to MS analysis after taking UV–Vis spectra. About 4 µl of the protein solution was directly injected into an LC-ESI-MS system (LC: Agilent 1290 Infinity II UPLC). A gradient from 15 to 80% acetonitrile in 0.1% formic acid (using a Waters BioResolve column (2.1 × 5 mm)) at a flow rate of 400 μl/min was applied (15 min gradient time). Detection was performed with a Q-TOF instrument (Agilent Series 6560 LC-IMS-QTOFMS) equipped with the Jetstream ESI source in positive ion, MS mode (range: 100–3,200 Da). Instrument calibration was performed using the ESI calibration mixture (Agilent Technologies). Data were processed using MassHunter BioConfirm B.08.00 (Agilent Technologies) and the spectrum was deconvoluted by MaxEnt.

### Time-Resolved UV–Vis Spectroscopy of Wild-Type *Cd*ChdC and Variants During Turnover

Pre-steady-state analysis of *Cd*ChdC and variants, reconstituted with coproheme in the presence of H_2_O_2_, was done by stopped-flow spectroscopy using a stopped-flow apparatus equipped with a photodiode array detector (SX-18MV; Applied Photophysics) or a monochromator (Pistar, Applied Photophysics). About 2–10 µM coproheme with a 1.5- to 2-fold surplus of protein was mixed with 200 µM H_2_O_2_ in 50 mM phosphate buffer pH 7.

### Spin-Trapping Experiments Using MNP of Wild-Type *Cd*ChdC and Y135AW183Y and W183Y Variants

2-Methyl-2-nitrosopropane (MNP) stock solution of 5 g ml^−1^ was prepared by dissolving MNP in H_2_O and heating it up to 60°C in the dark for 30 min. For a typical spin-trapping reaction, 30 µM coproheme reconstituted *Cd*ChdC was premixed with 0.5 g ml^−1^ MNP before adding H_2_O_2_ concentrations ranging from 0 to 2,400 µM. Spin trapping reaction mixes were prepared with a final volume of 100 μL, and the samples were analyzed by peptide mapping.

### Crystallization and Structure Refinement of the *Cd*ChdC Variant W183Y and Y135A Variant

For crystallization of *Cd*ChdC and variants, the vapor diffusion method was applied. A Mosquito LCP (TTP Labtech, Melbourn Science Park, Melbourn, United Kingdom) was used to set crystallization drops in SWISSCI 96-well 3-drop MRC crystallization plates (Molecular Dimensions, Newmarket, United Kingdom). Coproheme reconstituted and SEC purified protein stocks stored at −80°C were thawed, diluted with 50 mM phosphate buffer of pH 7, and supplemented with 10 mM NaCN to ∼6.8 μg μl^−1^ for pipetting in the crystallization plates. The reservoir was filled with 40 µl of precipitant solution and crystallization plates were stored in a Formulatrix RI-1000 imaging device at 22°C.

Preparation of seed solution from *Cd*ChdC was described previously (Michlits et al., 2020) and was performed analogously to the wild-type protein for Y135A. Single drops contained 150:200:100, 200:200:100, and 250:200:100, protein (nL):crystallization (nL):seed (nL). *Cd*ChdC Y135A crystallized with crystallization solution containing 18% PEG und 0.1 M MgCl. *Cd*ChdC W183Y was crystallized without seed, and single drops contained 150:200, 200:200, and 250:200, protein (nL):crystallization (nL). Diffracting crystals of W183Y could be collected from the Peg/Ion crystallization screen well H3 (20 %w/v PEG 3350 and 0.1 M CBTP 6.4 pH).

About 25% glycerol was supplemented to the crystallization solutions as a cryoprotectant for both variants. Crystals were collected from the crystallization drops with cryoloops and flash-vitrified in liquid nitrogen.

### Data Collection and Structure Refinement

Datasets were collected at the European Synchrotron Radiation Facility (ESRF, Grenoble, France) beamline ID23-2 (Flot et al., 2010) for W183Y at 100 K using a DECTRIS PILATUS3 X 2M and at ID29 (de Sanctis et al., 2012) for Y135A at 100 K using a DECTRIS PILATUS 6M. The dataset for W183Y was processed with XDS and symmetry equivalent reflections merged with XDSCONV (Kabsch, 2010). For Y135A, we used the data automatically processed with XDSAPP (Krug et al., 2012). Initially, we used a conservative high-resolution cutoff 2.27 Å (CC1/2 = 47.9; I/SIGMA = 1.53) for W183Y (Karplus and Diederichs, 2012). For both structures, the phase problem was solved by molecular replacement using phenix.phaser (McCoy et al., 2007) taking the wild-type protein structure (6XUC) as a template. The models were further improved by iterative cycles of the manual model building using COOT (Emsley et al., 2010) and maximum likelihood refinement using phenix.refine (Liebschner et al., 2019). For Y135A, phenix.refine converted intensities into amplitudes using the French and Wilson algorithm (French and Wilson, 1978), and the final high-resolution cutoff was based on performing paired refinement using the PDB_REDO webserver (Joosten et al., 2009). Final stages of refinement included Translation Liberation Screw (TLS) parameters, isotropic B-factor model, automated addition of hydrogens and water molecules, optimization of X-ray/ADP weight, and optimization of X-ray/stereochemistry weight. For W183Y, twinning was identified by PDB_REDO and phenix.xtriage and therefore refinement was performed using a twin law of -h,-k,l. The model was validated with MolProbity (Davis et al., 2004). Figures were prepared with PyMOL Molecular Graphics System (Version 1.3, Schrödinger, LLC). Atomic coordinates have been deposited in the Protein Data Bank under the accession code 7Q4G and 7Q4F.

## Results

### Variants Structural Features

**FIGURE 1:**
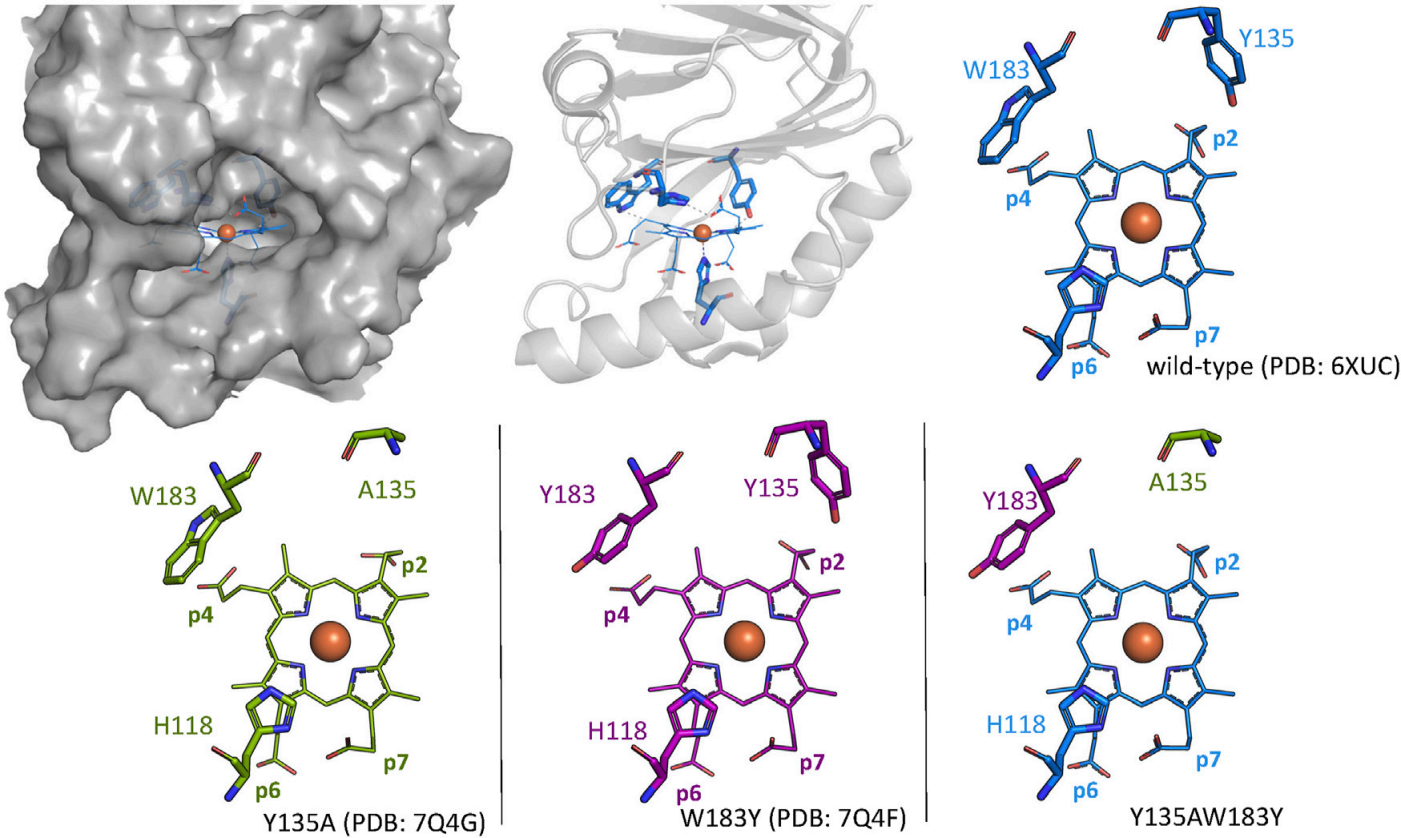
Wild-type *Cd*ChdC structure is shown as gray surface and cartoon representation. Active site structures of wild-type *Cd*ChdC (blue), Y135A (green), and W183Y (purple) are shown as stick figure representation. Crystal structure of the investigated Y135AW183Y variant could not be solved, and the expected active site structure is represented as a combination of the two single mutants from an overlay.

For this study, the single variant W183Y and double variant Y135AW183Y were investigated in order to assess their ability of catalyzing coproheme decarboxylation. A tyrosine residue is introduced as a potential radical site at the position of p4 in the active site in *Cd*ChdC W183Y. *Cd*ChdC Y135AW183Y lacks the original catalytic Y135. We hypothesized that radical formation in Compound I* formation is shifted to Y183. Crystal structures of the single variants Y135A (PDB: 7Q4G) and W183Y (PDB: 7Q4F) could be obtained, however screening for crystallization conditions for the double-variant Y135AW18Y was not successful (Figure 1; Table 1). The structures clearly show that the introduced amino acids occupy the positions of the respective residues in the wild-type protein. While the distance between Y135 and Cß-p2 is about 3.1 Å in the wild-type structure, the Y183 oxygen lies about 4.4 Å above the Cß of p4. Both single mutations leave the active site and the overall structure is unaffected.

**TABLE 1:** Data collection and refinement statistics^a^.

	*Cd*ChdC Y135A	*Cd*ChdC W183Y
Data collection		
Wavelength (Å)	0.8731	0.8731
Resolution range (Å)	48.31–1.818 (1.883–1.818)	43.509–2.150 (2.227–2.150)
Space group	P 1 21 1	P 3 1 2
Unit cell (Å)	61.117, 123.517, 78.3 90, 98.015, 90	141.89, 141.89, 124.94, 90, 90,120
Total reflections	659,863 (37,745)	1,548,284 (155,613)
Unique reflections	100,509 (8,240)	77,872 (6,892)
Multiplicity	6.6 (4.6)	19.9 (20.1)
Completeness (%)	97.47 (80.21)	96.95 (89.11)
Mean I/sigma(I)	5.72 (0.52)	9.37 (0.64)
Wilson B-factor (Å2)	27.41	44.08
Refinement		
R-merge (%)	0.1912 (1.847)	0.3783 (4.152)
R-meas (%)	0.2075 (2.089)	0.3883 (4.259)
R-pim (%)	0.07961 (0.95)	0.08721 (0.9445)
CC1/2	0.995 (0.216)	0.994 (0.223)
CC*	0.999 (0.596)	0.999 (0.603)
Reflections used in refinement	100,477 (8,241)	70,973 (3,777)
Reflections used for R-free	2,100 (172)	2,131 (109)
R-work	0.1734 (0.3619)	0.1395 (0.2491)
R-free	0.2194 (0.3892)	0.1822 (0.2905)
CC(work)	0.969 (0.542)	0.901 (0.391)
CC(free)	0.941 (0.482)	0.928 (0.308)
Number of nonhydrogen atoms	10,503	10,323
Macromolecules	9,410	9406
Ligands	442	269
Solvent	841	648
Protein residues	1,149	1,150
RMS(bonds) (Å)	0.015	0.00270
RMS(angles) (°)	0.97	1.16
Ramachandran favored (%)	97.89	97.37
Ramachandran allowed (%)	2.11	2.63
Ramachandran outliers (%)	0	0
Rotamer outliers (%)	0.42	0.94
Clashscore	3.33	3.28
Average B-factor (Å2)	35.54	48.00
Macromolecules (Å2)	35.41	
Ligands (Å2)	34.15	
Solvent (Å2)	37.39	
Number of TLS groups	33	40

a Statistics for the highest-resolution shell are shown in parentheses.

### Formation of Compound I in Wild-Type *Cd*ChdC and Variants

**FIGURE 2:**
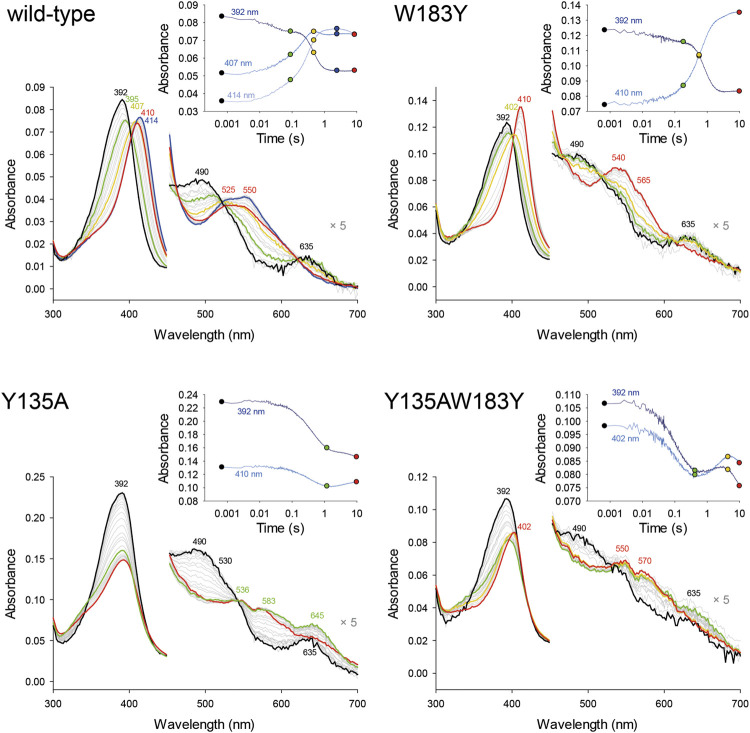
Pre-steady-state kinetics of wild-type *Cd*ChdC and variants W183Y, Y135A, and W183YY135A reaction with hydrogen peroxide was determined via stopped-flow spectroscopy. About 2–10 µM coproheme with a 1.5- to 2-fold surplus of protein were mixed with 200 µM H_2_O_2_ in 50 mM phosphate buffer of pH 7. Spectra at relevant time points are highlighted in black, green, yellow, or red, and intermediate spectra are shown in light grey. The insets show absorbance changes at indicated fixed wavelengths over the time and exact corresponding time points to the highlighted spectra are indicated as dots.

Pre steady-state kinetics of Compound I formation with H_2_O_2_ was determined by stopped-flow spectroscopy. Formation of Compound I is the precondition for the formation of Compound I* with a tyrosyl radical and subsequent decarboxylase activity. By comparing *k*_app_ of the wild-type and investigated variants (Table 2), it becomes apparent that the formation of Compound I is not affected by any of the introduced mutations and is in a range of 1.5–6.3 × 10^4^ M^−1^ s^−1^ (Figure 2).

**TABLE 2:** Pre-steady-state kinetic rate constants of compound I formation in *Cd*ChdC and variants.

	*k*_app_ (M^−1^s^−1^)	Source
Wild-type *Cd*ChdC	4.9 ± 1.3 × 10^4^	Michlits et al. (2020)
*Cd*ChdC Y135A	1.5 ± 0.2 × 10^4^	Michlits et al. (2020)
*Cd*ChdC W183Y	6.3 ± 0.6 × 10^4^	This study
*Cd*ChdC Y135AW183Y	6.5 ± 0.3 × 10^4^	This study

### Mass-Spectrometric Identification of Tyrosyl Radical Sites

**FIGURE 3:**
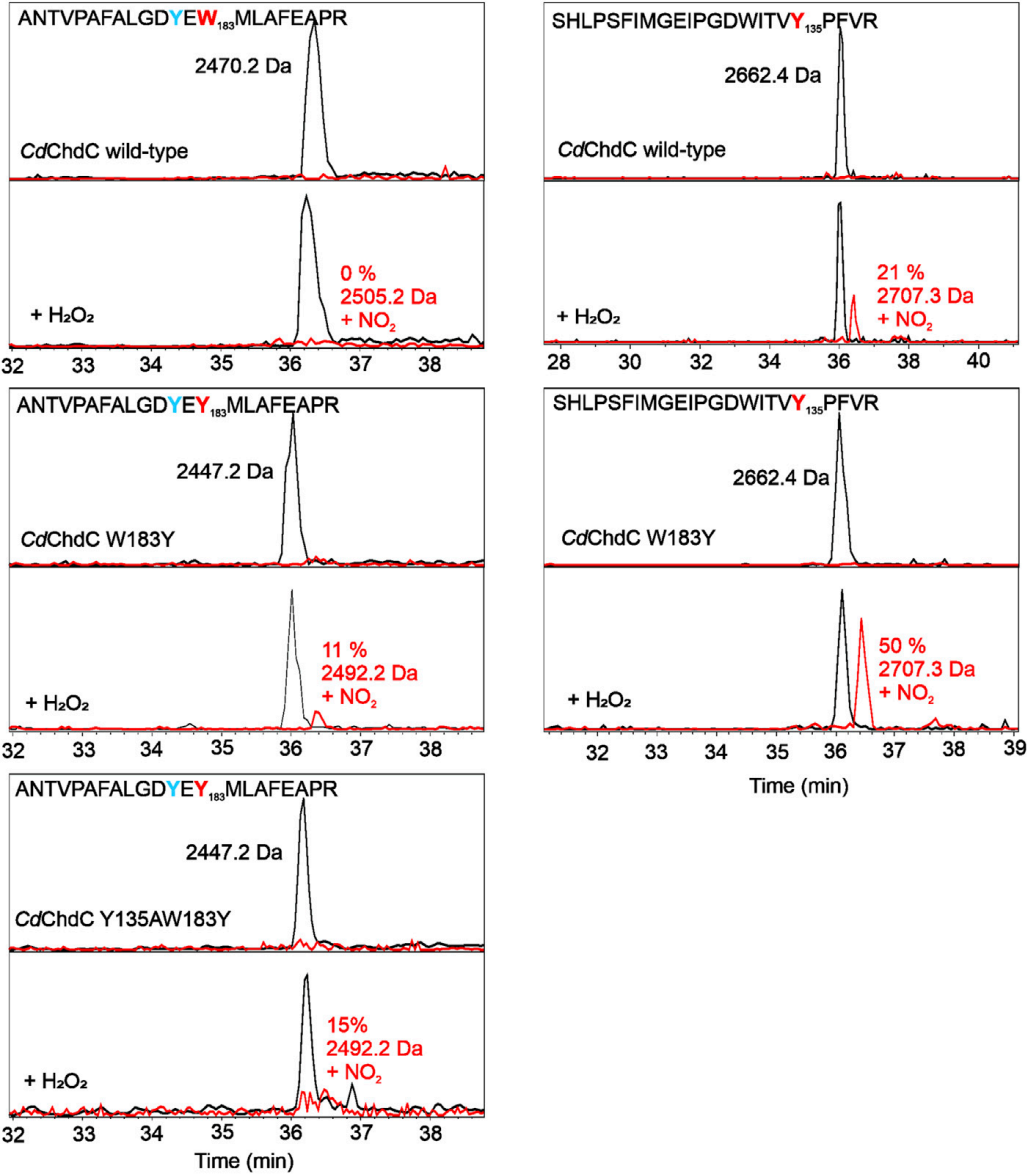
Identification of tyrosyl radicals in *Cd*ChdC and variants W183A and Y135AW183Y by MNP spin trapping. Mass spectrometric analysis of *Cd*ChdC with MNP without (top) and after addition of hydrogen peroxide (bottom). Peptides with an additional 46 Da (NO_2_) result from MNP modification of tyrosine radicals to nitrotyrosines.

MNP spin trapping and peptide mapping of wild-type *Cd*ChdC show no nitrosylation at the site of W183, where Y181 is also located, but after the addition of H_2_O_2_, 21% of the peptide comprising Y135 is nitrosylated (Figure 3). During the decarboxylation, Compound I and subsequent Compound I* formation leads to a specific radical site on the catalytic Y135. The introduction of another tyrosine in close proximity to the redox-active substrate leads to approximately 11% nitrosylation on Y183 and 50% radical formation on Y135. Compared to the wild-type protein this is even an increase in nitrosylated Y135, which can be explained by a hampered reaction and slight accumulation of Compound I* in the variant. In Y135AW183Y, elimination of the natural radical site Y135 and introduction of the artificial Y183 lead to the detection of about 15% nitrosylation on Y183, which is in fact comparable to the 21% Y135 nitrosylation in the wild-type protein, but Y183 radical formed in this variant appears to be incapable of decarboxylation as can be taken from mass spectrometric analysis of porphyrins in this variant upon titration with hydrogen peroxide (3.5).

### UV–Vis Absorbance Spectral Features of Wild-Type *Cd*ChdC and Variants During Turnover With H_2_O_2_

**FIGURE 4:**
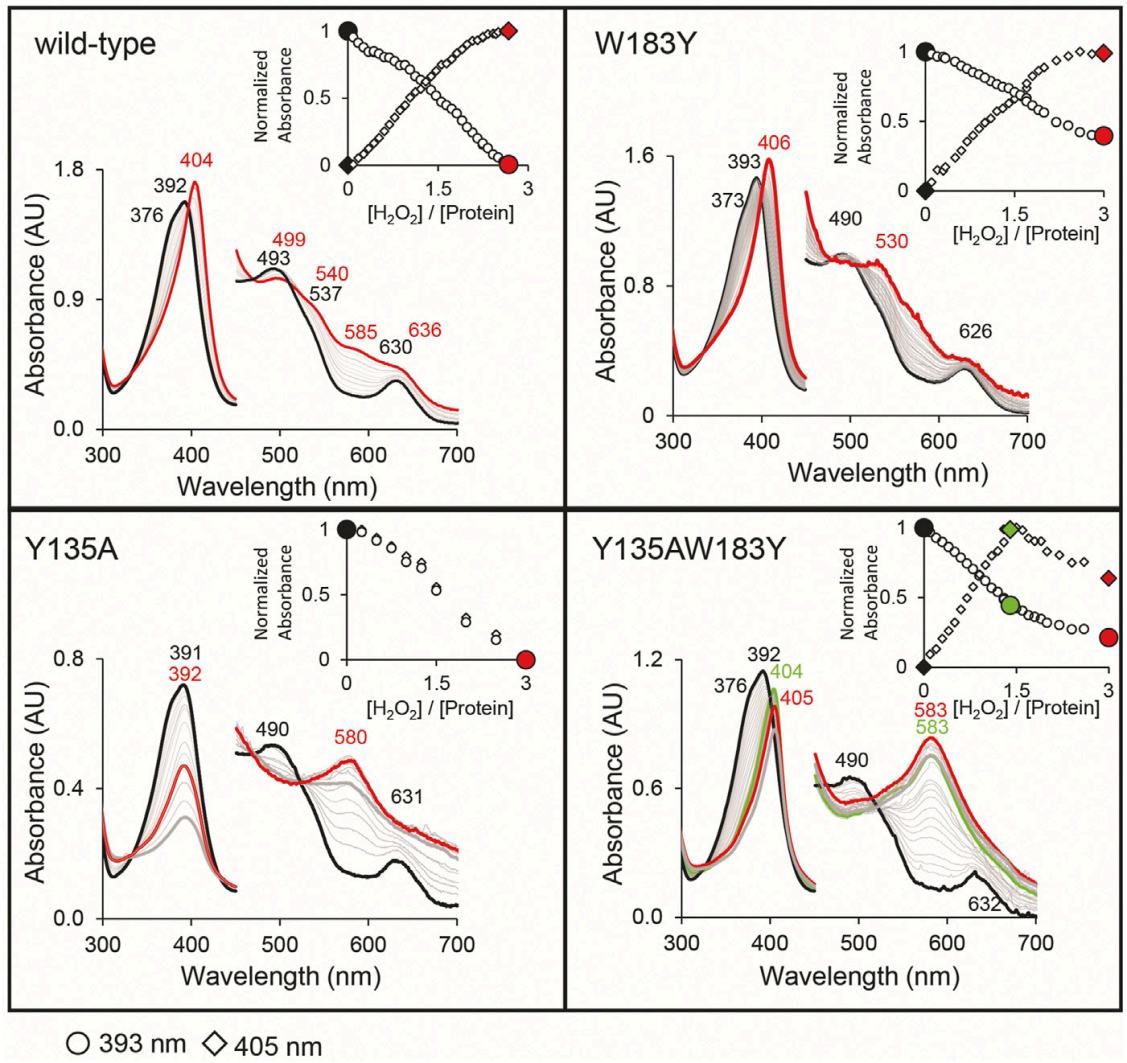
Decarboxylase activity of wild-type *Cd*ChdC and variants Y135A, W183Y, and Y135AW183Y with hydrogen peroxide, followed by UV–Vis spectroscopy. Spectral transition from the coproheme bound protein (red) upon addition of sub-equimolar amounts of H_2_O_2_. About 10–15 µM coporoheme bound to an 1.5 to 2-fold excess of protein were titrated with hydrogen peroxide in 0.1 molar equivalent steps in 50 mM phosphate buffer, pH 7. Insets show absorbance change at the coproheme Soret maximum (○) and the new maximum after the addition of three equivalents of hydrogen peroxide (◊).

Following UV–Vis absorbance spectral changes upon addition of hydrogen peroxide to the coproheme bound wild-type *Cd*ChdC, a clear shift from the resting state spectrum with a Soret maximum at 392 nm with a shoulder at 376 nm and visible bands at 493 and 630 nm to a new spectral species with a sharp Soret maximum at 404 nm and visible bands at 499, 540, and 636 nm was observed. This transition is completed after the addition of an approximate threefold excess of H_2_O_2_ (Figure 4). From complementary MS data, we know that we are observing the transition of the coproheme bound wild-type *Cd*ChdC via the MMD to the heme *b* bound protein (Michlits et al., 2020). Y135A, in contrast, exhibits a very similar UV–Vis spectrum to the wild type (Soret maximum at 391 with a shoulder, visible bands at 490 and 631 nm) but shows no spectral shift of the Soret maximum upon titration with H_2_O_2_. Instead, an absorbance decrease of the Soret maximum and emergence of a single band at 580 nm are observed (Figure 4). As there is a lack of catalytic Y135, no decarboxylation activity with H_2_O_2_ is observed in this variant (Michlits et al., 2020).

Introducing a second potential catalytic tyrosine in the W183Y variant, spectral features very similar to the wild-type protein are observable. While the characteristic shape of the *Cd*ChdC Soret maximum is slightly altered (393 nm with a less pronounced shoulder at 373 nm), the characteristic spectral shift of the Soret maximum upon addition of H_2_O_2_ is observed even to 406 nm. In the visible region, however, spectral features upon addition of a threefold excess of H_2_O_2_ appear different with maxima at 530 and 630 nm (Figure 4).

Most remarkably, in the double variant Y135AW183Y, which is lacking the physiological catalytic Y135 but contains an artificially introduced Y183, starting off from the coproheme reconstituted protein with spectral features almost identical to the wild type we observe a clear redshift of the Soret maximum upon titration with H_2_O_2._ A new spectral species with a Soret maximum at 404 nm (like in the wild type) and a strong band at 583 nm (like in Y135A) is observed with a 1.4-fold excess of hydrogen peroxide.

### Mass Spectrometric Semi-Quantification of Porphyrin Species During Turnover With H_2_O_2_

**FIGURE 5:**
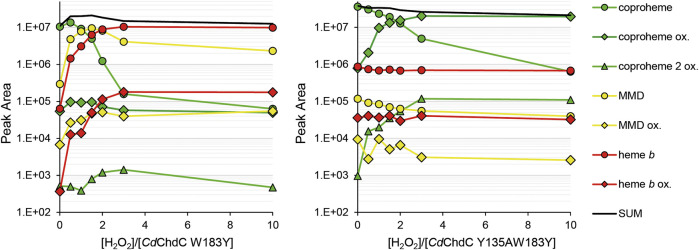
Semi-quantification of coproheme (green, 708.2 Da), MMD (yellow, 662.2 Da), and heme *b* (red, 616.2 Da) by mass spectrometry. Single oxidized species of the porphyrins are indicated as diamonds, and double oxidized coproheme is indicated as triangles. About 10–15 µM *Cd*ChdC W183Y and Y135AW183Y in complex with the coproheme in 50 mM phosphate buffer pH 7 were titrated with sub-equimolar amounts of hydrogen peroxide to monitor the course of the reaction. About 10 µl samples from the one 1 ml reaction mix were drawn and analyzed by mass spectrometry.

In our previous work, we showed wild-type *Cd*ChdC transforms supplied coproheme to MMD and subsequently heme *b* in the presence of H_2_O_2_ (Michlits et al., 2020). The transformation is completed with an approximately threefold excess of hydrogen peroxide. Y135A is unable to perform the decarboxylation reaction on coproheme. This variant will form Compound I in the presence of H2O2 and, due to the lack of the catalytic tyrosine for Compound I* formation, the variant will instead oxidize coproheme to get back to the ferric resting state. This is indicated by a 16 Da increase of the detected porphyrin masses in MS analysis. The mechanism of this observed oxidation, as well as its purpose, is still to be investigated. Introduction of a second tyrosine at the site of p4 in W183A slightly alters the UV–Vis spectral appearance of *Cd*ChdC and somewhat hampers decarboxylase activity by this intervention in the active site. The wild-type protein requires only 3 eq. H_2_O_2_ to complete the transformation of coproheme to heme *b*, and 30% remaining coproheme will be detected in W183Y. Y135AW183Y however shows no decarboxylation of coproheme whatsoever, only oxidation of the present porphyrin species upon addition of H_2_O_2_ is observed. MMD and heme *b*, oxidized or non-oxidized, are in a range of 104 m/z, and compared to the supplied coproheme and formed oxidized coproheme (up to 4 × 107 m/z) these can be considered minor traces from the purification (Figure 5).

## Discussion

In this study we attempted to engineer the active site of CdChdC to change its' stereospecificity. Instead of cleaving off p2 in the first step to yield 2-monovinyl-4-monopropionyl deuteroheme in the first step, we aimed to cleave off p4 first, in order to yield 2-monopropionyl-4-monovinyl deuteroheme (Scheme 1). Introduction of a tyrosine residue in proximity to p4, mimicking the situation with p2 and Y135, was implemented in the W183Y variant (Figure 1). Exchange of Y135 with a tryptophan, in order to mirror the situation in the active site, led to a very inactive variant (data not shown), we therefore eliminated Y135 by inserting an alanine residue (Y135A), as has proven to keep the formation of Compound I perfectly intact in previous studies (Michlits et al., 2020). We were able to show that in the double variant Y135AW183Y, tyrosyl radical formation in Compound I* is shifted to the artificial Y183 (Figure 3), however no altered decarboxylation activity could be observed in this variant compared to the wild type (Figure 4).

In the wild-type protein, the tyrosyl radical of Compound I* will immediately be neutralized by the proton-coupled electron transfer (PCET) from p2’s β-carbon and is thereby less efficiently “trapped” by MNP. Considering the fact that Y135AW183Y is unable to perform oxidative decarboxylation, it can be assumed that Compound I* will accumulate upon addition of hydrogen peroxide and should be readily detected. The comparable amounts of tyrosyl radical, detected in the wild type and Y135AW183Y, are therefore deceptive and Y183^•^ is in fact formed to a very lower extend than Y135^•^ in the wild type. This is also reasoned by the evaluation of the crystal structures and the assessment of the distance of the Y183 to the β-carbon of p4 (Figure 1). Nevertheless, minor amounts of formed Y183^•^-Compound I* are still unable to decarboxylate coproheme at p4, which leads us to the conclusion that the hydrogen bonding and spatial organization of the propionates in the active site are essential for ChdC decarboxylation reaction.

*Cd*ChdC active site appears to be perfectly optimized for electron channeling between the redox-active substrate and the catalytic Y135 for oxidative decarboxylation of p2 at this site (Zhang et al., 2020; Tian et al., 2021). In order to introduce a catalytic center at the site of p4, further potential residues are required to be exchanged in order to mirror the overall active site. Hampered formation of Y183^•^ further points to the fact that electron densities on the Compound I porphyryl radical are not equally distributed but will favor electron transfer from Y135, as a similarly focused distribution of a porphyryl radical has been reported for another heme protein (Linde et al., 2015).

Another possible route to rationally engineer *Cd*ChdC in order to produce 2-monopropionyl-4-monovinyl deuteroheme would be to intentionally alter the active site for coproheme binding in the reversed orientation (Aojula et al., 1987; Nagai et al., 2008; Rwere et al., 2008; Milazzo et al., 2020). In this way, p4 would initially be in close proximity to the catalytic Tyr135. Docking studies of 2-monovinyl-4-monopropionyl deuteroheme showed that, in principle, this possibility cannot completely be ruled out (Tian et al., 2021). However, due to the manifold factors that influence coproheme binding in ChdCs, the residues to be changed in order to obtain a reversed coproheme binding orientation are not straightforward to identify. This approach requires a concerted and systematic *in silico* study prior to wet lab experimental work. At the same time, *in silico* calculations of electron migrating pathways to follow up the results presented in this study will be crucial for assessment if the rational design approach to alter ChdC’s active site for stereospecific production of the three-propionate intermediates is a fruitful one.

## Data Availability

The datasets presented in this study can be found in online repositories. The names of the repository/repositories and accession number(s) can be found in the article/Supplementary Material.
